# Long-term effect of transurethral partial cystectomy with a 2-micrometer continuous-wave laser for non-muscle-invasive bladder cancer

**DOI:** 10.3389/fsurg.2023.1117997

**Published:** 2023-04-17

**Authors:** Yongliang Lu, Sinan Jiang, Xiaotao Yin, Jiaxiang Guo, Xiaoying Zhu, Han Ma, Guohui Zhang, Hualiang Yu, Yi Xiao, Yong Yang

**Affiliations:** ^1^ Senior Department of Urology, The Third Medical Centre of PLA General Hospital, Beijing, China; ^2^ Department of Urology, The Fourth Medical Centre of PLA General Hospital, Beijing, China

**Keywords:** long-term effect, 2um laser, cystectomy, bladder cancer, non-muscle invasive

## Abstract

**Purpose:**

We have reported the efficacy and safety of 2-micrometer continuous-wave laser cystectomy of non-muscle invasive bladder tumor (NMIBC) (J Urol. 2009;182:66–9). In this study, we evaluated the long-term outcomes of patients with NMIBC who underwent transurethral partial cystectomy with a 2-micrometer continuous-wave laser, and explored the risk factors for tumor recurrence.

**Methods:**

This was a retrospective study of patients with NMIBC planned to undergo transurethral partial cystectomy with a 2-micrometer continuous-wave laser at the Fourth Medical Center of the PLA General Hospital between January 2012 and December 2014. The primary outcome was bladder cancer recurrence.

**Results:**

A total of 75 patients were enrolled. Sixty-two (82.7%) were male. The patients were 59.8 ± 12.9 years of age. The mean operation time was 38.7 ± 20.4 min. No Clavien grade >2 complications occurred. The duration of catheter indwelling was 3.6 ± 1.8 days. The hospital stay was 6.0 ± 2.3 days. The median follow-up was 80 months. A total of 17 patients had a recurrence during follow-up, and the recurrence-free survival (RFS) rate was 77.3%. In the multivariable analysis, the tumor risk group were independently associated with the recurrence of NMIBC (*p* = 0.026).

**Conclusions:**

After TURBT with a 2-micrometer continuous-wave laser, RFS was 77.3% at the median follow-up of 80 months. All complications were mild. Only tumor risk group was independently associated with the recurrence of NMIBC.

## 1. Introduction

Bladder cancer (BC) incidence, diagnosed in both sexes combined, ranks 10th place with 573,278 estimated number of incident cases worldwide in 2020, which has been on the rise annually with the onset of aging (1). BC can be classified into non-muscle-invasive BC (NMIBC) and muscle-invasive BC (MIBC) based on the depth of invasion, and approximately 75% of newly detected cases are non–muscle-invasive disease (2–5). Advanced age, male sex, and cigarette smoking contribute to the development of bladder cancer. BC mainly occurs in persons >55 years of age, with a median of 73 years, and it is 3–5 times more frequent in men than in women (2, 4–7). The age-standardized incidence of BC is 9.5 per 100,000 men and 2.4 per 100,000 women (1). The exact causes are unknown, but the development of BC is likely multifactorial and may include a combination of environmental factors, chronic bladder irritation, and genetic factors. The most important risk factor is tobacco smoke (active and passive) (3–5, 8).

NMIBC is defined as stage Ta, T1, or carcinoma *in situ* (CIS) disease (4). Transurethral resection of bladder tumor (TURBT) is recommended for the primary evaluation and initial treatment of NMIBC (2–4). The goal of TURBT is to resect all visible tumors completely (i.e., R0 resection) and to determine the clinical stage and grade of the disease (2). The use of classical TURBT may eradicate stage Ta-T1 tumors, but they commonly recur and progress to MIBC. In addition, a persistent disease after initial resection is found in 33%–55% of patients with T1 tumors and in 41% of patients with Ta grade 3 tumors (4). Those patients may undergo a second TURBT or surgery, but the survival benefit of a salvage procedure is controversial (9, 10). In addition, each session of TURBT is associated with a risk of complications such as bleeding, obturator nerve reflex, and perforation (11). Therefore, the removal of all lesions during the first TURBT might improve survival.

Laser ablation was developed to improve the efficacy and safety of TURBT (12). Lasers without deep penetration cause less pain and bleeding, and the power of the laser can be adjusted according to the extent of the lesions. Available lasers include the neodymium laser, yttrium aluminum garnet laser, potassium titanyl phosphate laser (green-light laser), holmium laser, and 2-micrometer continuous-wave laser (thulium laser) (12). The holmium laser has been suggested to be a better option than conventional TURBT in patients with NIMBC (12–14). The 2-micrometer continuous-wave laser, first used for NMIBC in 2009 by our team (15), is also safe and effective for smooth incision, tissue vaporization, and *en bloc* resection of NMIBC. Nevertheless, prospective trials of laser TURBT and with a long follow-up are still lacking regarding the 2-micrometer continuous-wave laser (12, 16).

Therefore, the aim of the present study was to examine the long-term outcomes of patients with NMIBC who underwent TURBT with the 2-micrometer continuous wave laser and to explore the risk factors for tumor recurrence.

## 2. Materials and methods

### 2.1. Study design and subjects

This was a prospective study of consecutive patients with NMIBC planned to undergo TURBT with a 2-micrometer continuous-wave laser at the Fourth Medical Center of the PLA General Hospital between January 2012 and December 2014. This study was approved by the Ethics Committee of the Fourth Medical Center of the Chinese PLA General Hospital. Written informed consent was obtained from all subjects.

The inclusion criteria were: (1) patients planned to undergo TURBT with a 2-micrometer continuous-wave laser; (2) patients with postoperative pathological diagnosis of NMIBC (Tis, Ta, or T1); (3) first onset of disease; and (4) expected life expectancy after surgery of >5 years. The exclusion criteria were: (1) any other incurable malignant tumors; (2) history of pelvic radiotherapy; (3) history of bladder surgery; (4) extensive tumor lesions of the bladder, which could not be completely removed by a single surgery; or (5) follow-up was impossible.

### 2.2. Surgical methods and postoperative management

**Figure 1:**
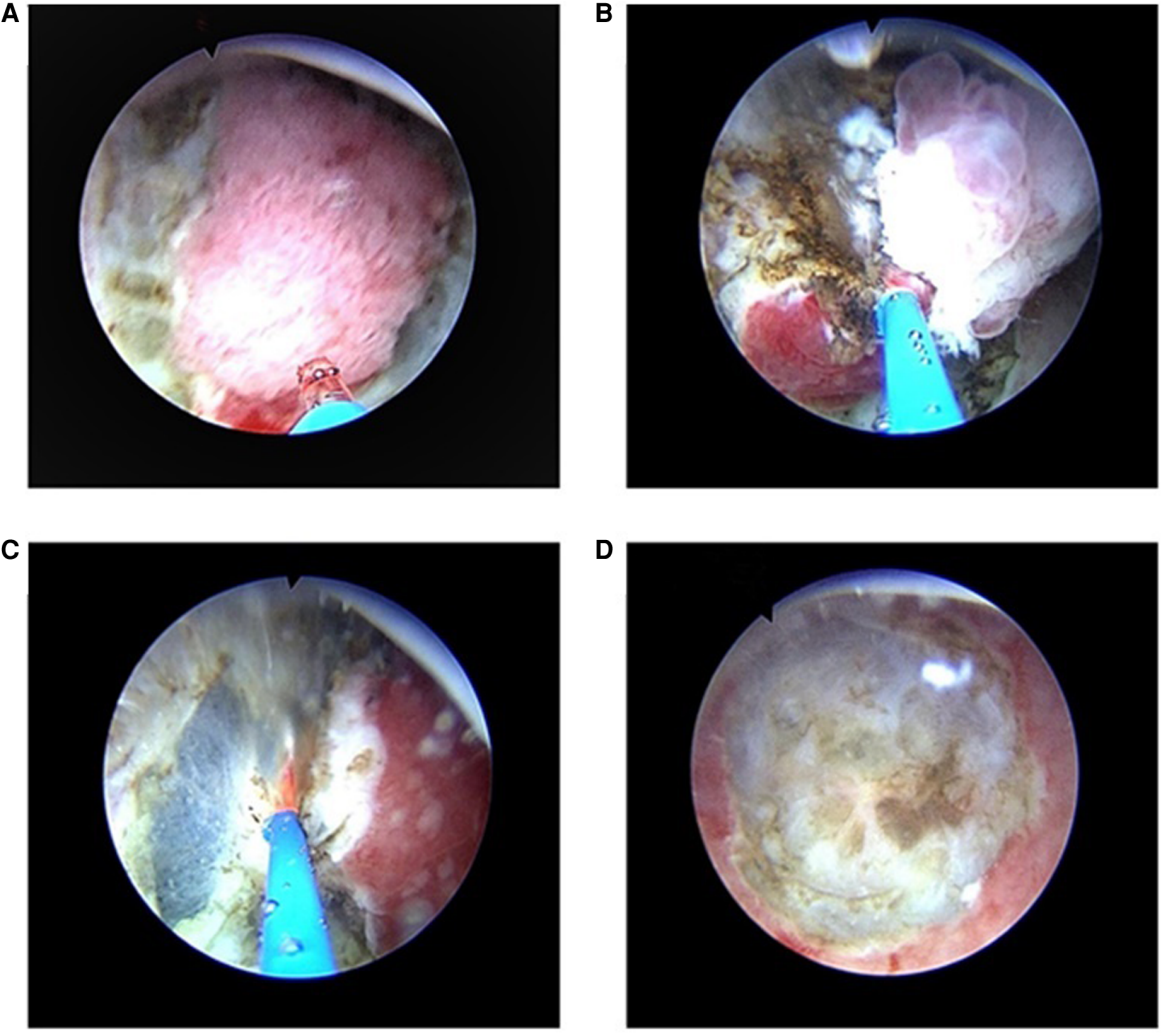
TURBT with a 2-micrometer continuous-wave laser for NIMBC (**A**) First, the normal mucosa and submucosa about 0.5-cm–1.0-cm away from the tumor were vaporized and incised. (**B**) If the tumor was large, the superficial tumors were segmentally removed, and then the whole muscle layers at the tumor base were peeled in a layered and segmented manner. (**C,D**) If the tumor was small, the muscular and serosal layers at the tumor base were directly removed, and the tumor, basal part, and part of the muscle layer underwent *en bloc* resection.

For the TURBT, the RevoLix 2-µm continuous-wave medical laser surgery system produced by the LISA Company (Germany) was used, with a laser wavelength of 2.01 µm. The energy was transmitted through a 600-µm optical fiber, and the power was set to 50–60 W. A laser resectoscope (China Hawkmed Company, Beijing, China) was used with a 26.5 Fr catheter. The surgical procedures were performed under a television monitoring system. Epidural or general anesthesia was conducted, and the patient was placed in the lithotomy position. Intraoperatively, 0.9% saline was used as the rinse solution. After the laser resectoscope was inserted, the bladder tumor was examined in terms of size, number, morphology, and location. The optical fiber-probe was sent to the bladder through the operation channel of the laser resectoscope, and the position of the laser spot was adjusted after starting up. First, the normal mucosa and submucosa about 0.5-cm–1.0-cm away from the tumor were vaporized and incised (Figure 1A). If the tumor was large, the superficial tumors were segmentally removed (Figure 1B). Then, the whole muscle layers at the tumor base were peeled in a layered and segmented manner. If the tumor was small, the tumor, basal part, and part of the muscle layer underwent *en bloc* resection (Figures 1C,D). After the postoperative indwelling of the catheter for 3–5 d, the bladder washout was determined by the color of the drainage urine. According to tumor size, resection extent, and pathologic conditions, intravesical infusion chemotherapy was performed on the day of surgery or within 1 week. A second TURB was performed within two-six weeks after initial resection in the following situations: after incomplete initial TURB, or in case of doubt about completeness of a TURB; if there is no muscle in the specimen after initial resection, with the exception of TaLG/G1 tumors and primary CIS; in T1 tumors.

### 2.3. Data collection and definitions

Demographic data (age and sex), tumor site, pathological grading, and staging were collected. Intraoperative and postoperative data included operation time, intraoperative and postoperative complications, special intraoperative treatment, duration of catheter indwelling, and hospital stay. The risk groups were divided into a low-risk group, intermediate group, and high-risk group using the standard of the European Association of Urology (EAU).

### 2.4. Follow-up

Follow-up was performed using outpatient clinic visits and telephone. The primary outcome was the recurrence of the BC. The recurrence-free survival (RFS) was defined as the length of time from operation to tumor recurrence. All patients underwent the first cystoscopy 3 months after surgery. If the patients in the low-risk group had negative results for the first cystoscopy, a second cystoscopy was performed in the first year after surgery, and once every year until the 5th year thereafter. The patients in the intermediate- and high-risk groups underwent cystoscopy every 3 months for the first 2 years. From the 3rd year, the cystoscopy was performed every 6 months; from the 5th year, it was performed once every year for life. The follow-up ended in December 2020 or tumor recurrence.

### 2.5. Statistical analysis

All data were analyzed using SPSS 22.0 (IBM, Armonk, NY, United States). All continuous variables were tested for normal distribution using the Kolmogorov–Smirnov test. Data that conformed to the normal distribution were presented as means ± standard deviations. Data not conforming to the normal distribution were presented as medians (ranges). A Kaplan–Meier curve was drawn for the RFS of the patients, and the log-rank test was used for comparison of the curves. A Cox multivariable analysis was performed with disease recurrence as the dependent variable; the results were presented as hazard ratio (HR) and 95% confidence interval (CI). Two-sided *p*-values <0.05 were considered statistically significant.

### 2.6. Patient and public involvement

There was no patient or public involvement in any aspect of this study or its write-up.

## 3. Results

### 3.1. Characteristics of the patients

A total of 75 patients were enrolled (Table 1). Sixty-two (82.7%) patients were male. The patients were 59.8 ± 12.9 years of age. The maximum tumor size was 2.1 cm ± 0.8 cm, with 64 (85.3%) patients with stage Ta BC and 11 (14.7%) with stage T1 BC. Ten (13.3%) cases were papillary urothelial neoplasm of low malignant potential (PUNLMP), 56 (74.7%) cases were low-grade BC, and nine (12.0%) were high-grade BC. According to the EAU risk stratification, 43 (57.3%) patients were at low risk, 18 (24.0%) were at intermediate risk, and 14 (18.7%) were at high risk.

**Table 1:** Characteristics of the patients.

Variables	Patients (*n* = 75)
Sex (Male), *n* (%)	62 (82.7)
Age (years)	59.8 ± 12.9
Smoking history, *n* (%)	28 (37.3)
**Tumor site, *n* (%)**	
Lateral wall	65 (86.7)
Posterior wall	5 (6.7)
Anterior wall	7 (9.3)
Triangle area	7 (9.3)
Bladder neck	7 (9.3)
Maximum diameter of the tumor (cm)	2.1 ± 0.8
**Number of tumors, *n* (%)**	
Solitary tumors	61 (81.3)
Multiple tumors	14 (18.7)
T-stage, *n* (%)	
Ta	64 (85.3)
T1	11 (14.7)
**Grade, *n* (%)**	
PUNLMP	10 (13.3)
Low	56 (74.7)
High	9 (12.0)
**Risk group (EAU), *n* (%)**	
Low	43 (57.3)
Intermediate	18 (24.0)
High	14 (18.7)

PUNLMP, papillary urothelial neoplasm of low malignant potential; EAU, european association of urology.

### 3.2. Intraoperative and postoperative characteristics of the patients

Table 2 presents the intraoperative and postoperative data of the patients. The mean operation time was 38.7 ± 20.4 min. No Clavien grade 2 or above complications occurred. The duration of catheter indwelling was 3.6 ± 1.8 days. The hospital stay was 6.0 ± 2.3 days.

**Table 2:** Intraoperative and postoperative data.

Variables	Patients (*n* = 75)
Time of operation (min)	38.7 ± 20.4
Intraoperative complications	0
Intraoperative special treatments	
Implantation of ureteral stents	4
Dilation of urethra	1
Postoperative complications	No complications of Clavien grade 2 or above
Duration of catheter indwelling (days)	3.6 ± 1.8
Hospital stay (days)	6.0 ± 2.3
Follow-up (months)	80 (3–107)

### 3.3. Recurrences

**Figure 2:**
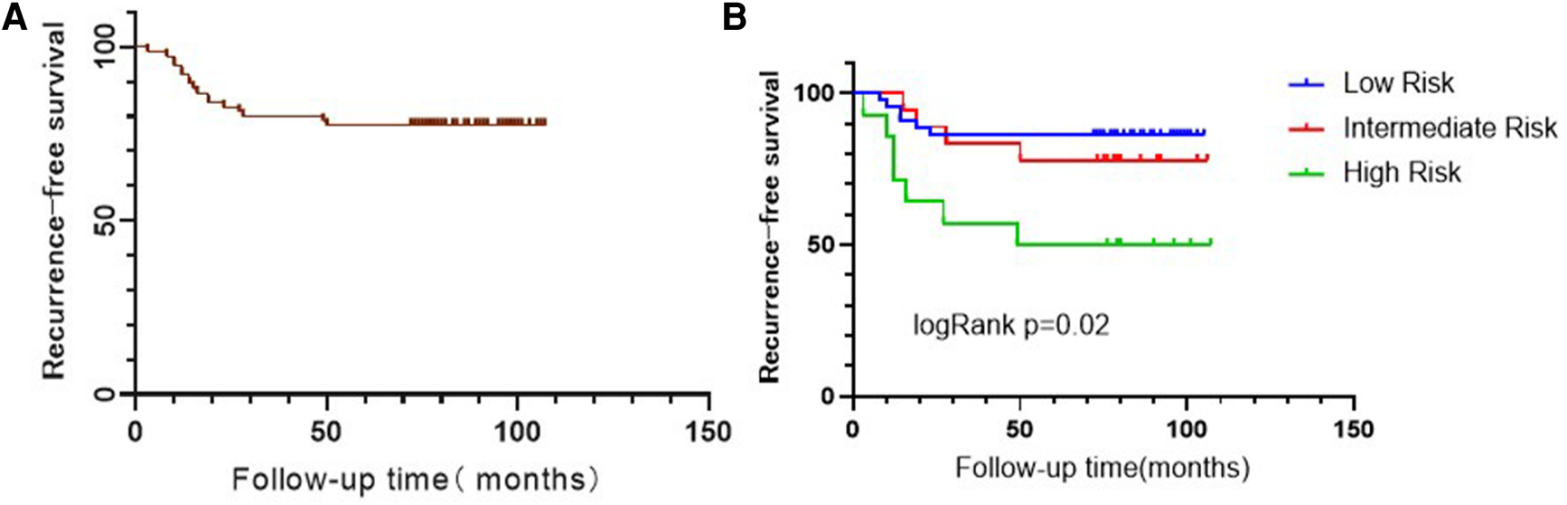
Kaplan-Meier curve of recurrence-free survival in all patients (**A**) and in different risk group (**B**).

A total of 17 patients had a recurrence, and the RFS rate was 78.7% at median follow-up of 80 months (range, 3–107 months). Figure 2 presents the Kaplan-Meier curve of RFS. The 1- and 3-year recurrence rates were 8% (6/75) and 20% (15/75), respectively. The specific recurrence rates were 15.6% (10/64) and 63.6% (7/11) for stage Ta and T1 BC, respectively; 10.0% (1/10), 21.4% (12/56), and 44.4% (4/9) for PUNLMP, low-grade, and high-grade BC, respectively; and 14.0% (6/43), 22.2% (4/18), and 50% (7/14) for low, intermediate and high-risk group, respectively (Table 3).

**Table 3:** The recurrence rate of different subgroups of patients.

Variables	Patients (*n* = 17)
**T-stage, *n* (%)**	
Ta (*n* = 64)	10 (15.6)
T1 (*n* = 11)	7 (63.6)
**Grade, *n* (%)**	
PUNLMP (*n* = 10)	1 (10.0)
Low (*n* = 56)	12 (21.4)
High (*n* = 9)	4 (44.4)
**Risk group (EAU), *n* (%)**	
Low (*n* = 43)	6 (14.0)
Intermediate (*n* = 18)	4 (22.2)
High (*n* = 14)	7 (50.0)

In the multivariable analysis (Table 4), the tumor risk groups were independently associated with the recurrence-free survival of NMIBC (intermediate risk group, HR = 0.271, 95% CI: 0.087–0.843, *p* = 0.024; high risk group, HR = 0.199, 95% CI: 0.052–0.766, *p* = 0.019).

**Table 4:** Multivariable analysis of recurrence-free survival.

Variables	Multivariable analysis	95% CI	*p*
	HR		
Sex			0.960
Age			0.231
Smoking history			0.345
Risk			**0** **.** **026**
Low	Reference		
Intermediate	0.271	0.087–0.843	**0**.**024**
High	0.199	0.052–0.766	**0**.**019**
Maximum diameter of the tumor			0.111
Multiple tumors	3.326	1.000–11.064	0.050

HR, hazard ratio; CI, confidence interval.

Bold values indicate significant difference at *p* < 0.05.

## 4. Result

Prospective trials of transurethral partial cystectomy for NMIBC are still lacking, especially regarding the long-term outcomes of the 2-micrometer continuous wave laser. Therefore, the aim of this study was to examine the long-term outcomes and to explore the risk factors for tumor recurrence. The results from the 75 patients suggest that RFS at a median follow-up time of 80 month after transurethral partial cystectomy is 77.3% and that the complications are mild. The tumor risk group and multiple tumors were independently associated with the recurrence of NMIBC.

The 2-micrometer continuous wave laser was invented and first used for NMIBC in 2009 by Yang et al. (15), who showed that it could be used for the treatment of NMIBC in nine patients. More recent studies showed that the operation time is around 25–48 min, depending upon tumor size and number of lesions (17–20). In the present study, the operation time was 38.7 ± 20.4 min, falling within the reported range. Regarding complications, a meta-analysis of five studies by Bai et al. (12) showed that the overall complication rate of the TURBT with a 2-micrometer continuous wave laser was 1.4% and that none were life-threatening, supporting the present study. Huang et al. (21) reported that no complication occurred among 70 patients treated with the 2-micrometer continuous wave laser. Similar results were observed by Li et al. (22) in 136 patients.

Our first study of the 2-micrometer continuous-wave laser for NMIBC did not assess recurrence (15). Liu et al. (23) compared TURBT with the 2-micrometer continuous wave laser and conventional TURBT in 60 patients, and reported no difference in recurrences between the two groups, with 10.9%, 19.5%, and 31.3% at 1, 2, and 3 years for the 2-micrometer continuous-wave laser, and 10.7%, 22.9%, and 33.9% for the conventional approach. Another study by the same authors, with 120 patients this time, showed that the 1-year recurrence rate after treatment with the 2-micrometer continuous-wave laser (12.0%) was lower than with conventional TURBT (17.2%) and the holmium laser (25.0%) (17). Zhong et al. (24) (*n* = 97) observed similar 2-year recurrence rates between the 2-µm laser and the holmium laser (26.7% and 24.0%), both lower than with TURBT (31.0%). Zhang et al. (18) reported a 3-year recurrence rate of 45.6% among 149 patients who received 2-micrometer continuous-wave laser TURBT. After a mean follow-up of 41 months, Li et al. (22) reported an overall recurrence rate of 68%. In the present study, the recurrence rate with median follow-up of 80 months is 22.7% among 75 patients, which is much lower than in the previous studies presented above. The exact reason for this result is unknown but could be due to the transurethral partial cystectomy regarding the 2-micrometer continuous wave laser, or to the sample size, the learning curve, the patient population/selection criteria. In 2016, Huang et al. (21) reported a 2-year recurrence rate of 10.9% among 70 patients treated with the 2-micrometer continuous-wave laser, which could support the low rate observed in the present study.

In the present study, only tumor risk group was independently associated with recurrence, with 14.0%, 22.2%, and 50% for the low, intermediate, and high-risk groups, respectively. This is supported by results by Zhang et al. (18), who showed recurrence rates of 14.7%, 42.1%, and 62.5% for low, intermediate, and high-risk groups, respectively, but the rates in the study by Zhang et al. (18) were higher than in the present study. On the other hand, Li et al. (22) reported no significant differences among the three risk groups (62%, 65%, and 71%). Again, this could be due to the sample size, the learning curve, or to the patient population.

Of course, the present study has limitations. The sample size was from a single institution and was relatively small. In addition, there was no comparator group. Differences in the recurrence rate could hint toward a bias from the learning curve or the incision of tumors with full-thickness detrusor muscle layers at the base and around the tumors, which could be further explored in future studies.

## 5. Conclusion

In conclusion, the present study suggests that the recurrence rate with median follow-up of 80 months after transurethral partial cystectomy with a 2-micrometer continuous-wave laser was 22.7%, representing a good curative effect. The tumor risk group was independently associated with the recurrence of NMIBC. All complications were mild. However, retrospective analysis and prospective cohort study would be conducted to verify the curative effect. In view of the above, this surgical treatment may be expected to play an important role in bladder-preserving tri-modality therapy for patients with MIBC.

## Data Availability

The raw data supporting the conclusions of this article will be made available by the authors, without undue reservation.
